# Correlation Between the Proportion of Senescence-Associated β-Galactosidase-Stained CD8+ T Cells and Age: A Cross-Sectional Study in Japan

**DOI:** 10.3390/ijms26188799

**Published:** 2025-09-10

**Authors:** Masaya Tsubokawa, Yoshiki Shimizu, Misato Yazaki, Shieri Shimodan, Masayuki Noguchi, Arisa Yamazaki, Tomomichi Watanabe, Makoto Ocho, Tsuyoshi Sakurada, Yoshie Hirose, Jiro Saito, Yuri Ishii

**Affiliations:** 1 FANCL Research Institute, Yokohama 244-0806, Japan; tsubokawa_masaya@fancl.co.jp (M.T.); misato_110401@fancl.co.jp (M.Y.); shieri2104@fancl.co.jp (S.S.); noguchi_masayuki@fancl.co.jp (M.N.); arisa2004@fancl.co.jp (A.Y.); watanabe_tomomichi@fancl.co.jp (T.W.); ocho_makoto@fancl.co.jp (M.O.); sakurada_tsuyoshi@fancl.co.jp (T.S.); yuishii@fancl.co.jp (Y.I.); 2 Ginza Yoshie Clinic, Yukeikai Medical Corporation, V88 Building 5F, 2-5-11 Ginza, Chuo-ku, Tokyo 104-0061, Japan; yh@ginzabiyou.com; 3 Medical Station Clinic, 3F Ichikawa Gakugei-dai Building, 3-12-8 Takaban, Meguro-ku, Tokyo 152-0004, Japan; j.saito@med-station.jp

**Keywords:** age, senescence, senescence-associated β-galactosidase, CD8+ T cells

## Abstract

Recently, senescent T cells in the peripheral blood have been detected using senescence-associated β-galactosidase (SA-βGal) activity and have been used as an endpoint in clinical trials. However, the epidemiological association between the abundance of SA-βGal-stained senescent CD8+ T cells and chronological age has not been fully elucidated. To examine the correlation between the proportion of SA-βGal^high^ CD8+ T cells and age, we analyzed previously collected clinical trial data. We conducted a cross-sectional analysis of 632 Japanese adults aged 40–59 years who participated in the screening phase of a clinical trial. To characterize senescent CD8+ T cells, we measured the proportion of SA-βGal^high^ in total CD8+ T cells and each subset—naïve, central memory (TCM), effector memory (TEM), and terminally differentiated effector memory (TEMRA). We then calculated the correlation coefficients between the proportion of SA-βGal^high^ CD8+ T cells and age. The proportion of SA-βGal^high^ cells in total CD8+ T cells, naïve, TCM, TEM, and TEMRA CD8+ T cells increased significantly with age. In Japanese adults, the proportion of SA-βGal^high^ in CD8+ T cells may serve as a useful biomarker of immune senescence.

## 1. Introduction

In modern society, medical advances are increasing average life expectancy, resulting in an aging world population. By 2050, the proportion of the world’s population aged ≥65 years may reach more than 1.5 times that of the current level [1]. In an aging society, a reduction in physiological function increases the number of elderly people with frailty [2,3], thereby increasing the risk of mortality and need for nursing care [3,4]. Aging progresses in biological, psychological, and social domains [5], and the risk of age-related diseases increases based on environmental factors as well as biological factors such as immune senescence [6,7]. As the world population ages, preventing the decline of age-associated physiological functions can extend its lifespan.

Assessing the degree of aging is crucial for its prevention and management. Chronological age—calculated from the date of birth—can be used to assess aging because of its strong association with health deterioration, morbidity, and mortality [8,9]. However, aging is a heterogeneous process, and individuals of the same chronological age have considerable variations in health status. Therefore, chronological age is considered an inadequate reflection of biological function [8,9]. Additionally, when assessing the anti-aging effects of an intervention, the extension of life expectancy or chronological age alone may be insufficient for clinical assessment, as the intervention may enhance healthy life expectancy without significantly affecting survival [10]. Therefore, it is crucial to develop biomarkers that reflect diverse biological functions as indicators of the biological aging process. Biological aging is characterized by the decline in cellular and organ functions, with cellular senescence being one primary contributing factor. Cellular senescence is characterized by irreversible cell cycle arrest that cannot be restored by stimulation. This distinguishes it from temporary cell cycle arrest—where proliferation resumes upon appropriate stimuli—and terminal differentiation that involves permanent cell cycle arrest and results in the acquisition of specific cellular functions [11,12]. Quantifying the abundance of senescent cells may enable the measurement of cellular senescence and assessment of biological aging.

Senescence-associated β-galactosidase (SA-βGal) is often used as a biomarker for cellular senescence because of the increased activity of lysosomal β-galactosidase in senescent cells at pH 6.0, where no enzymatic activity is observed in normal cells [13]. SA-βGal staining is gaining attention as an early screening method for cellular senescence because SA-βGal activity increases with age in various tissues [14]. Notably, SA-βGal is easily detected both in vivo and in vitro [15], making it a valuable biomarker for cellular senescence associated with age-related diseases [16]. Additionally, recent advances have enabled the detection, quantification, and isolation of senescent T cells in peripheral blood mononuclear cells (PBMCs) using a fluorogenic and self-immobilizing substrate of SA-βGal as an indicator of immune senescence [17]. Although the immune system plays a crucial role in maintaining homeostasis and protecting the body from foreign pathogens, the senescence of immune cells induces a proinflammatory senescence-associated secretory phenotype (SASP) in vivo [18]. Among immune cells, CD8+ T cells play a role in the adaptive immune system by controlling malignancy and intracellular infection, and the accumulation of senescent CD8+ T cells with enhanced proinflammatory potential leads to senescence and contributes to the progression of various age-related diseases [19,20]. Furthermore, senescent CD8+ T cells have been reported to predict the development of hyperglycemia in humans and have also been used to assess diabetic patients in clinical trials [21,22], potentially expanding their role as a biomarker of immune senescence.

Senescent T cells in PBMCs detected through SA-βGal activity have also been associated with senescence and have been assessed as an endpoint in recent clinical trials for immune senescence [23]. Previous studies have suggested that the abundance of SA-βGal-stained senescent CD8+ T cells in PBMCs differs between young and elderly individuals [17]. However, there have been no epidemiological studies examining the association between SA-βGal-stained senescent CD8+ T cells and age in middle-aged individuals. Furthermore, there is a lack of research into the association between the proportion of SA-βGal^high^ CD8+ T cells within each CD8+ T cell subset and age. Therefore, we analyzed the correlation between the proportion of SA-βGal^high^ CD8+ T cells and chronological age using large-scale data from participants in the screening phase of a clinical trial assessing the effects of plant extract on immune senescence markers.

## 2. Results

Table 1 presents the clinical characteristics of the study cohort. The cohort included 303 males (48.0%) and 329 females (52%), with a mean age and body mass index (BMI) of 50.2 years and 21.9 kg/m^2^, respectively. The blood test parameters were generally normal, and none of the participants had severe disease. The blood parameters other than low-density lipoprotein cholesterol were worse in males than in females.

Table 2 presents the characteristics of CD8+ T cells in lymphocytes. In all participants, the mean proportion of CD8+ T cell subsets was high for naïve (34.6%) and low for terminally differentiated effector memory T cells (TEM) re-expressing CD45RA (TEMRA) (13.9%) cells. The average proportion of SA-βGal^high^ expression in the total CD8+ T cells was 65.5%. TEM in the CD8+ T cell subsets was significantly higher in males. When analyzed by each CD8+ T cell subset, it was low in naïve (10.1%), intermediate in central memory T cells (TCM; 53.1%), and high in TEM (93.2%) and TEMRA (96.3%) cells. The proportion of CD8+ TEM was higher in males than in females, but the proportion of SA-βGal^high^ in each CD8+ T cell subset did not differ by sex. We provide box plots for the proportion of each CD8+ T cell subset in all CD8+ subsets and SA-βGal^high^ in each CD8+ T cell subset in Figure S1. In addition, the mean ± SD of total CD4+ in lymphocytes was 52.9 ± 8.74% and SA-βGal^high^ in total CD4+ was 52.3 ± 17.78%, with no sex difference observed.

Table 3 presents the correlation coefficient between age and each CD8+ T cell subset in lymphocytes. Total CD8+ T and naïve CD8+ T cell levels significantly decreased with age, whereas TCM, TEM, and TEMRA cell levels significantly increased. Among the correlations between CD8+ T cell subsets and age, the correlation between naïve CD8+ T cells and age was relatively moderate (Pearson’s r = 0.308, *p* < 0.001; Spearman’s r = 0.320, *p* < 0.001). The proportion of SA-βGal^high^ in total CD8+ T cells, naïve, TCM, TEM, and TEMRA CD8+ T cells significantly increased with age. Among the correlations between SA-βGal-stained senescent CD8+ T cells and age, the correlation between the proportion of SA-βGal^high^ in total and age was relatively moderate (Pearson’s r = 0.300, *p* < 0.001; Spearman’s r = 0.314, *p* < 0.001). In the sex-based subgroup analysis, there was no significant correlation between TEMRA CD8+ cell levels and age in males, whereas total CD8+ T cells, along with TCM and age, demonstrated no significant correlation in females. However, significant correlations were observed for each of the other CD8+ T cells and age, consistent with the overall analysis. Notably, the proportion of SA-βGal^high^ in total CD8+ T cells, naïve, TCM, TEM, and TEMRA CD8+ T cells significantly increased with age, even in the sex-based subgroup analysis. On the other hand, no significant correlation was observed between the proportion of SA-βGal^high^ in total CD4+ T cells and age (Pearson’s r = 0.072, *p* = 0.071; Spearman’s r = 0.058, *p* = 0.146). Similarly, no significant correlation was observed in the subgroup analysis by sex (Pearson’s r = 0.05, *p* = 0.387; Spearman’s r = 0.042, *p* = 0.464 and Pearson’s r = 0.093, *p* = 0.094; Spearman’s r = 0.074, *p* = 0.183 for male and female, respectively).

**Figure 1:**
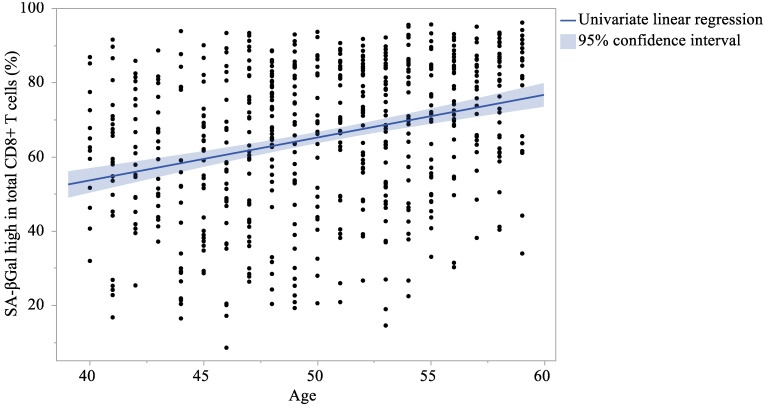
Correlation between the proportion of SA-βGal^high^ in total CD8+ T cells and age.

**Figure 2:**
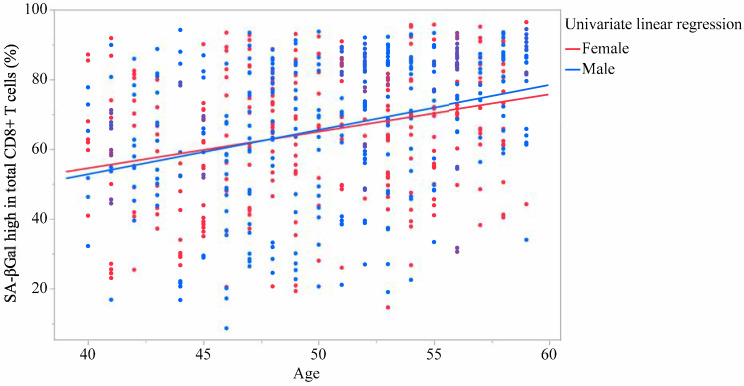
Correlation between the proportion of SA-βGal^high^ in total CD8+ T cells and age by sex.

Table 4 presents the results of the linear regression analysis with each CD8+ T cell as the response variable and age as an explanatory variable. The multivariate analyses were adjusted for sex and BMI. Similar to univariate analysis, the multivariate analysis demonstrated that the total CD8+ T cell and naïve CD8+ T cell levels were significantly reduced with age, whereas TCM, TEM, and TEMRA CD8+ T cell levels were significantly increased. Moreover, the proportion of SA-βGal^high^ in total CD8+ T cells, and naïve, TCM, TEM, and TEMRA CD8+ T cells significantly increased with age. Figure 1 and Figure 2 showed linear univariate regression analyses of the correlation between the proportion of SA-βGal^high^ in total CD8+ T cells and age. Figure S2 showed linear univariate regression analyses of the correlation between the proportion of SA-βGal^high^ in each CD8+ T cell subset and age. On the other hand, no significant regression coefficient was observed between the proportion of SA-βGal^high^ in total CD4+ T cells and age (β = 0.245; 95%CI = −0.021 to 0.511; *p* = 0.071 and β = 0.245; 95%CI = −0.02 to 0.51; *p* = 0.07, for univariate and multivariate, respectively).

Table 5 presents the correlation coefficient between the proportion of SA-βGal^high^ in total CD8+ T cells and each CD8+ T cell subset in lymphocytes. Increases in the proportion of SA-βGal in total CD8+ T cells correlated with decreases in naïve T cells and increases in TEM and TEMRA. Only the Spearman correlation coefficients showed a significant correlation between the increase in the proportion of SA-βGal^high^ in total CD8+ T cells and the increase in TCM.

Table 6 presents the results of the linear regression analysis with the proportion of SA-βGal^high^ in total CD8+ T cells as the response variable and each CD8+ T cell subset as an explanatory variable. The multivariate analyses were adjusted for age, sex, and BMI. The multivariate analysis demonstrated that naïve CD8+ T cell and TCM levels were significantly decreased with an increase in the proportion of SA-βGal^high^ in total CD8+ T cells, whereas TEM and TEMRA T cell levels were significantly increased. Linear univariate regression analyses of the correlation between the proportion of SA-βGal^high^ in total CD8+ T cells and each CD8+ T cell subset are shown in Figure S3.

## 3. Discussion

In our study, the proportion of SA-βGal^high^ in total CD8+ T cells increased significantly with age in Japanese males and females. Age-related increases in the proportion of SA-βGal^high^ were also evident in each CD8+ T cell subset examined—naïve, TCM, TEM, and TEMRA. To our knowledge, no epidemiological study has measured SA-βGal-stained senescent CD8+ T cells in a population as large as that in our study. In a small study comparing donors in their 20s and 60s, the proportion of SA-βGal^high^ in total CD8+ T cells increased in older donors compared to that in younger donors; however, significant associations were not confirmed, except in TEMRA T cells analyzed using CD8+ T cell subsets [17]. In this study, the abundance of SA-βGal-stained senescent CD8+ T cells varied greatly depending on the type of CD8+ T cell subset, and the proportion of SA-βGal^high^ in CD8+ had a wide range of values. Our findings support their previous findings, revealing significant correlations not only between the proportion of SA-βGal^high^ in total CD8+ T cells but also in each CD8+ T cell subset. In their previous study, they found that the proportion of SA-βGal^high^ in total CD4+ T cells was also higher in people in their 60 s compared with those in their 20 s, but our study did not find any significant correlation between the proportion of SA-βGal^high^ in total CD4+ T cells and age. This difference in results is thought to be because our study is limited to participants in their 40s and 50s, and because CD4+ T cells are less affected by age than are CD8+ T cells. In fact, a previous study also confirmed that the degree of increase in SA-βGal^high^ with age in total CD4+ T cells was lower than that in the proportion of SA-βGal^high^ with age in total CD8+ T cells [17]. The fact that CD8+ T cells are more affected by the aging process than are CD4+ T cells and that the properties of CD8+ T cells change with age is consistent with previous findings on immune cell senescence [24,25].

Furthermore, our data is not only consistent with these previous findings but also shows that senescent cells accumulate in undifferentiated areas with age, extending observations that had previously been inconclusive. To date, there has been limited conclusive evidence regarding the characteristics of cellular senescence in human naïve CD8+ T cells with respect to age [17], but this study showed that SA-βGal-stained senescent cells increase even within naïve CD8+ T cells with age. Naïve CD8+ T cells are T cells that are not activated until they first encounter a specific antigen. They play a key role in the early stages of the adaptive immune response. Previous studies have shown that the naïve CD8+ T cell compartment shrinks with age, and the increased clonality and decreased priming efficiency of naïve CD8+ T cells have also been reported in elderly individuals [26,27]. Also, recent epigenetic studies have reported that aged naïve CD8+ T cells displayed a loss in chromatin accessibility at the gene promoters [28], and that levels of SA-βGal^high^ activity also defined changes in chromatin accessibility [29]. Among the T cell subsets, TEMRA CD8+ T cells are also known to be potential indicators of immune senescence and exhibit high cytotoxicity and proinflammatory capacity [30]. In this study, SA-βGal-stained senescent CD8+ T cells were more nicely correlated with age than TEMRA CD8+ T cells; thus, measuring the proportion of SA-βGal^high^ in total CD8+ T cells is useful. In summary, taken together with the data from this study, these results suggest that the proportion of SA-βGal^high^ in total CD8+ T cells may serve as a senescence indicator that is somewhat independent of differentiation.

Regarding the biological significance of the abundance of SA-βGal-stained senescent CD8+ T cells, it has been reported that CD8+ T cells with SA-βGal^high^ activity exhibit telomere dysfunction-induced senescence and p16^INK4a^-mediated senescence and express certain SASP genes, such as tumor necrosis factor (TNF) and interferon (IFN) [17]. P16^INK4A^ inhibits cell proliferation and suppresses tumors [31], while its prolonged expression induces cellular senescence [32]. Telomere dysfunction induces a DNA damage response and is therefore considered one of the physiological triggers of age in vivo [33]. TNF-α and IFN-γ are inflammatory substances induced in vivo by T cells with mitochondrial dysfunction owing to mitochondrial transcription factor A deficiency [34,35]. Recent epigenetic studies have also reported that SA-βGal^high^-expressing CD8+ T cells transition to a dysfunctional state, which may contribute to autoimmune disorders and various age-related diseases [29].

Moreover, we showed that the proportion of SA-βGal^high^ was very low in naïve CD8+ T cells, and the proportion of SA-βGal^high^ in total CD8+ T cells was correlated with naïve CD8+ T cells even after adjusting for age. Based on our findings, the proportion of SA-βGal^high^ in CD8+ T cells may be associated with the function of CD8+ T cell subsets. T cells have diverse differentiation phenotypes [36], and immune senescence is characterized by a reduction in naïve CD8+ T cells and an increase in memory T cells [37,38]. CD8+ T cell subsets are crucial in immune senescence studies because TEMRA CD8+ T cells may represent a type of senescent T cell [20,30], and naïve CD8+ T cells exhibit a younger epigenetic age than effector memory CD8+ T cells in the same individual [39]. Indeed, it has been shown that the expression levels of various surface, functional, and molecular markers involved in cellular senescence differ among each T cell subset [40]. Regarding the clinical significance of naïve CD8+ T cells, it has been suggested that levels of naïve CD8+ T cells may be a prognostic indicator for oligometastatic non-small cell lung cancer patients and are reduced in patients with chronic kidney disease (CKD) [41,42]. SA-βGal^high^ expression also correlates with increased cellular malignancy, indicating that SA-βGal may be used in the pathological diagnosis of tumors and treatment assessment [43]. Cellular senescence is also associated with diabetes and CKD, and SA-βGal expression increases in the renal tubular compartment of CKD [44,45]. Recently, it has been reported that the inhibition of sodium-glucose cotransporter 2, which is useful for treating diabetes and CKD, reduces SA-βGal activity in visceral adipose tissue and attenuates pathological aging [46]. Because SA-βGal-stained tissues in the liver and kidney and SA-βGal-stained senescent CD8+ T cells were also observed to increase in aged mice [47], targeting the abundance of SA-βGal-stained senescent CD8+ T cells may be useful in diagnosing and treating age-related diseases.

In addition, we showed that the proportion of SA-βGal^high^ was very high in TEM and TEMRA CD8+ T cells, and the proportion of SA-βGal^high^ in total CD8+ T cells was correlated with TEM and TEMRA CD8+ T cells even after adjusting for age. Because CD57 and Killer cell lectin-like receptor subfamily G, which are often used as senescent T cell markers, also show similar trends [48], it is highly likely that SA-βGal-stained CD8+ T cells can also be used as a marker of senescent CD8+ T cells. Senescent T cells accumulate in the nervous system with age and contribute to the onset and progression of neurodegenerative diseases [49], as well as the cognitive decline often associated with Alzheimer’s disease (AD) [50]. TEMRA CD8+ T cell accumulation may occur in response to neuronal damage or neuroinflammation owing to aging and may be associated with the onset of Parkinson’s disease (PD) in addition to the progression of AD [51,52]. TEMRA CD8+ T cells may be a minimally invasive biomarker that can be used for the early diagnosis of idiopathic PD in females because their accumulation is more prevalent in females than in males during the early to intermediate stages of idiopathic PD [53]. Females are more susceptible to various autoimmune diseases [54], and TEMRA CD8+ T cell accumulation has been indicated as a potential contributor to autoimmune responses [53]. Because the proportion of SA-βGal^high^ in TEMRA CD8+ T cells is very high, sex differences should be carefully observed when using SA-βGal-stained senescent CD8+ T cells in PBMCs as biomarkers of immune senescence.

According to our results, the proportion of SA-βGal^high^ cells in total CD8+ T lymphocytes increased with age to a similar extent in both males and females. When analyzing individual subsets, sex-dependent differences emerged. Age only showed a positive correlation with TEMRA CD8+ cells in females, while age-related increases in total and TCM T cells were limited to males. However, because each CD8+ T cell level significantly correlated with age in the sex-adjusted analysis, we determined that sex differences had a minimal effect on each CD8+ T cell’s correlation with age. On the other hand, CD8+ TEM T cell levels were higher in males than in females. This sex difference is consistent with previous reports, and it may explain the difference in healthy life expectancy between males and females [37]. The influence of sex hormones as a mechanistic driver for immune senescence is a plausible hypothesis because it has been suggested that the immune response is enhanced by estrogen and suppressed by androgens/progesterone [55]. Similarly, males over 65 typically exhibit enhanced innate/inflammatory responses and impaired adaptive responses [56]. These data emphasize the necessity of considering sex differences in mechanistic studies and clinical applications of immune senescence indicators. Large-scale, preferably longitudinal cohort studies targeting the elderly are essential for clarifying how sex-dependent immune senescence pathways contribute to aging and its clinical outcomes.

The strengths of this study are as follows. Although SA-βGal has been traditionally used to stain tissues in patients, our study observed that the number of SA-βGal-stained CD8+ T cells in PBMCs increased with age. Cellular senescence has various phenotypes and cell states based on the cell type and physiological state, making it challenging to reliably quantify senescent cells. However, immune cells in the blood are suitable biological samples for assessing senescence because of their ease of isolation and analysis as cell types. While measurement requires a fluorescence-activated cell sorting (FACS) system and specialized technicians, it only necessitates standard PBMC separation and fluorescent SA-βGal probes, making implementation in clinical laboratories feasible through the establishment of standardized protocols. The evidence from our study population supports using the proportion of SA-βGal^high^ T cells in total CD8+ T cells as a reliable indicator for evaluating immune senescence, making them suitable for stratifying participants in clinical trials and monitoring disease risk. On the other hand, assessing SA-βGal^high^ levels in CD8+ T cell subsets provides insights into specific T cell senescence. In fact, recent clinical studies have focused on measuring various biomarkers of cellular senescence in CD8+ T cell subsets to assess the degree of senescence [57]. Approaches such as this that target key cellular senescence associated with aging and tissue degeneration have great anti-aging potential [58].

Limitations of our study include the cross-sectional study design, single-ethnic population, lack of functional assays (cytotoxicity and cytokine release), strictness of FACS measurement principles, and potential batch effects in SA-βGal staining. Because this assessment was a cross-sectional study of a general population in their 40s and 50s, we could not confirm its usefulness for the functional aspects of senescence or response to longevity interventions. Future epidemiological studies and intervention trials in elderly populations, including patients with age-related diseases, are anticipated to assess the association between SA-βGal-stained CD8+ T cells in PBMCs and molecular biomarkers of aging or biological age [59,60]. Furthermore, although there are various potential cellular senescence markers [61], this study specifically measured only SA-βGal-stained senescent CD8+ T cells as a response biomarker of immune senescence. Moving forward, it is necessary to verify causality through response analysis in longevity intervention studies, reproducibility in multi-ethnic populations, and targeted analysis with various biomarkers of biological function to establish a clinically reliable immune senescence indicator.

## 4. Materials and Methods

### 4.1. Participants and Analysis

This study analyzed screening test data from a clinical trial conducted in Japan (UMIN000051574). The cross-sectional analysis included 632 cases in which PBMCs were collected from 635 male and female participants (aged 40–59 years) who underwent screening for the clinical trial. This study included general males and females who did not suffer from serious illnesses such as autoimmune disease or cancer. This study was approved by the Medical Station Clinic Research Ethics Committee (approval number: 241128-1) and adhered to the principles of the Declaration of Helsinki. Written informed consent was obtained from all participants [23].

### 4.2. Clinical Features

All clinical tests were conducted in the morning while the participants were fasting. The blood parameters analyzed included blood glucose, hemoglobin A1c, total cholesterol, high-density lipoprotein, low-density lipoprotein, triglycerides, aspartate transaminase, alanine transaminase, gamma-glutamyl transaminase, urea nitrogen, and creatinine. Blood samples were collected from the peripheral veins and analyzed at LSI Medience (Tokyo, Japan). BMI was calculated from weight and height (kg/m^2^). Systolic and diastolic blood pressures were measured using an automated monitor while the participants were seated at rest [23].

### 4.3. PBMC Collection and Preservation

To collect PBMCs, blood was collected from the participants using BD Vacutainer^®^ CPT™ mononuclear cell preparation tubes with sodium citrate (BD Biosciences, Franklin Lakes, NJ, USA). Subsequently, the samples were centrifuged at 1800× *g* to separate PBMCs. The PBMC layer was then collected in a 15 mL tube, resuspended in phosphate-buffered saline (PBS) (-) (Thermo Fisher Scientific, Inc., Waltham, MA, USA), and centrifuged again at 400× *g*. After removing the supernatant, the cells were resuspended in Bambanker^®^ medium (Nippon Genetics, Inc., Tokyo, Japan) and stored at −80 °C [23].

### 4.4. Flow Cytometric Analysis of PBMCs

Cell staining was performed as described previously [17], with the following modifications. Bafilomycin A1 and SPiDER-βGal from the Cellular Senescence Detection Kit, SPiDER-βGal (Dojindo Laboratories, Kumamoto, Japan), were dissolved in 30 and 20 μL of dimethyl sulfoxide (FUJIFILM Wako Pure Chemical Corporation, Osaka, Japan), respectively. Cryopreserved 5 × 10^5^ human PBMCs were thawed and washed with FACS buffer (PBS [-], 2% fetal bovine serum [*v*/*v*] [Thermo Fisher Scientific, Inc.], 2 mM ethylenediaminetetraacetic acid [Thermo Fisher Scientific, Inc.], and 1% penicillin-streptomycin [Sigma Aldrich, St. Louis, MO, USA]), and treated with bafilomycin A1 (diluted 1:500 in Hank’s balanced salt solution (HBSS) [-]) (FUJIFILM Wako Pure Chemical Corporation) at 37 °C for 1.5 h [23]. βGal is expressed regardless of senescence. It is widely known that βGals are primarily localized in lysosomes, and that βGal is active near acidic pH 4.0, whereas overexpressed βGal in senescent cells (SA-βGal) is active even near neutral pH 6.0. SPiDER-βGal reacts with all βGal, but specific detection of SA-βGal was improved by inhibiting lysosomal acidification with the addition of the ATPase inhibitor Bafilomycin A1.

PBMCs were treated with SPiDER-βGal and bafilomycin A1 (diluted 1:500 in HBSS [-]) at 37 °C for 30 min, washed with FACS buffer, and treated with human TruStain FcX™ (BioLegend, San Diego, CA, USA, diluted 1:100) on ice for 20 min. For characterization of cell surface receptors, PBMCs were incubated on ice for 30 min with PE-conjugated anti-human CD3 (SK7, 1:100), Brilliant Violet 510™-conjugated anti-human CD4 (SK3, 1:100), PerCP/cyanine5.5-conjugated anti-human CD8a (RPA-T8, 1:100), Alexa Fluor^®^ 647-conjugated anti-human CD45RA (HI100, 1:5000), Brilliant Violet 421™-conjugated anti-human CD197 (CCR7) (G043H7, 3:100), and PE/cyanine7-conjugated anti-human CD279 (programmed cell death protein 1 [PD-1]) antibodies (EH12.2H7, 1:100), while with Alexa Fluor^®^647-conjugated Mouse IgG2b, κ Isotype Ctrl (MPC-11, 1:50,000), Brilliant Violet 421™-conjugated Mouse IgG2a, κ Isotype Ctrl (MOPC-173, 21:500), and PE/cyanine7-conjugated Mouse IgG1, κ Isotype Ctrl (MOPC-21, 1:100) (all from BioLegend) as isotype controls. After washing with FACS buffer, the PBMCs were treated with Fixable Viability Dye eFluor™ 780 (Thermo Fisher Scientific, Inc., 1:1000) on ice for 20 min and analyzed using a BD FACS Celesta instrument (BD Biosciences) and FlowJo software v10.8.1 (FlowJo, LLC, Ashland, OR, USA). PBMCs were acquired at 100,000 events for flow cytometry analysis [23].

CD8+ subsets were identified using isotype controls with the following markers:

- Naïve T cells: CD197 (CCR7)+CD45RA+;
- TCM: CD197 (CCR7)+CD45RA-;
- TEM: CD197 (CCR7)-CD45RA-;
- TEMRA: CD197 (CCR7)-CD45RA+;
- PD-1-positive cells: CD279+;
- T cells with SA-βGal^high^ expression.

CD8+ T cells are classified into naïve, TCM, TEM, and TEMRA cells. To quantify the proportion of T cells expressing high levels of SA-βGal, we leveraged the fact that donors often had separate cell populations with low and high SA-βGal fluorescence. Specifically, we set a gating threshold at the intersection of the two populations, defining SA-βGal^low^ and SA-βGal^high^ as populations with low and high signal intensities, respectively [23]. Additionally, our flow cytometry gating strategy is shown in Supplementary File S2.

### 4.5. Statistical Analysis

Continuous variables for the clinical and CD8+ T cell characteristics of the participants are presented as the mean ± standard deviation. Student’s *t*-test and Wilcoxon signed-rank test were conducted to determine sex differences in laboratory values.

To assess age-correlated CD8+ T cell characteristics, both Pearson’s and Spearman’s correlation coefficients between each CD8+ T cell in lymphocytes (%) and age were calculated in the overall and sex subgroups. For linear regression analysis, linear models were fitted to continuous response data using least squares estimation. The computational algorithm aimed to minimize the sum of squares of the differences (residuals) between the predicted and observed values of the response variable. Univariate linear regression analysis was used to calculate the effect of age for each CD8+ T cell, with each CD8+ T cell as the response variable and age as the explanatory variable. Multivariate linear regression analysis was used to calculate the effect of age on each CD8+ T cell by adjusting for sex and BMI. Each CD8+ T cell was used as the response variable, and age, sex, and BMI were used as explanatory variables. For reference, we also confirmed the correlation between the proportion of SA-βGal^high^ in total CD4+ T cells and age using a similar method.

In addition, to confirm the correlation between SA-βGal-stained senescent CD8+ T cells and CD8+ T cell differentiation, both Pearson’s and Spearman’s coefficients between the proportion of total CD8+ SA-βGal^high^ and the proportion of each CD8+ T cell subset were calculated in the overall and sex subgroups. Univariate linear regression analysis was used to calculate the effect of each CD8+ T cell subset on the proportion of SA-βGal^high^ in total CD8+ T cells, with the proportion of SA-βGal^high^ in total CD8+ T cells as the response variable and each CD8+ T cell subset as the explanatory variable. Multivariate linear regression analysis was used to calculate the effect of each CD8+ T cell subset on the proportion of SA-βGal^high^ in total CD8+ by adjusting for age, sex, and BMI. The proportion of SA-βGal^high^ in total CD8+ T cells was used as the response variable, and each CD8+ T cell subset, age, sex, and BMI were used as explanatory variables.

The regression coefficient (β) was calculated using the standard least-squares method, and a *t*-test was performed against the null hypothesis stating that the β parameter is zero. Statistical significance was set at *p* < 0.05 in two-tailed tests, and the 95% confidence interval for β was calculated. All statistical analyses were performed using the JMP^®^ Ver. 18 software (SAS Institute, Inc., Cary, NC, USA).

## 5. Conclusions

In our study, the proportion of SA-βGal^high^ in total CD8+ T cells and naïve, TCM, TEM, and TEMRA cells increased significantly with age in Japanese males and females. Our findings indicate that SA-βGal-stained senescent CD8+ T cells in PBMCs are useful as a minimally invasive and highly reliable biomarker for immune senescence. SA-βGal^high^ T cells in total CD8+ T cells capture both differentiation status and intrinsic cellular senescence, suggesting that it is a more comprehensive indicator of immune senescence than subset counting alone. Additionally, the proportion of SA-βGal^high^ in each CD8+ T cell subset that increased with age indicates that SA-βGal activity may be used as a response biomarker for specific T cell senescence. Further studies are required to clarify their usefulness as predictive or surrogate biomarkers of immune senescence.

## Figures and Tables

**Table 1:** Clinical characteristics in the study cohort.

		All	(N = 632)	Male	(N = 303)	Female	(N = 329)	*t*-Test	Wilcoxon
Variables	(Unit)	Mean	(SD)	Mean	(SD)	Mean	(SD)	*p*	*p*
Age	(years)	50.2	(5.22)	50.2	(5.22)	50.2	(5.23)	0.987	0.912
BMI	(kg/m^2^)	21.9	(3.22)	22.9	(3.19)	20.9	(2.92)	<0.001	<0.001
HbA1c	(%)	5.4	(0.31)	5.4	(0.34)	5.4	(0.28)	0.350	0.965
Blood glucose	(mg/dL)	87.0	(8.31)	87.7	(8.79)	86.5	(7.81)	0.071	0.016
Triglyceride	(mg/dL)	101.4	(65.99)	114.4	(75.50)	89.5	(53.21)	<0.001	<0.001
Total cholesterol	(mg/dL)	218.9	(35.69)	213.1	(34.17)	224.2	(36.27)	<0.001	<0.001
HDL cholesterol	(mg/dL)	71.6	(19.10)	64.2	(17.30)	78.4	(18.16)	<0.001	<0.001
LDL cholesterol	(mg/dL)	123.9	(30.85)	123.4	(29.90)	124.4	(31.74)	0.694	0.868
ALT	(U/L)	19.9	(13.13)	22.8	(13.88)	17.3	(11.81)	0.197	0.010
AST	(U/L)	21.7	(9.37)	22.2	(8.17)	21.3	(10.34)	<0.001	<0.001
γ-GTP	(U/L)	32.0	(49.89)	38.8	(40.52)	25.8	(56.52)	0.001	<0.001
Urea nitrogen	(mg/dL)	13.6	(3.70)	14.1	(3.62)	13.1	(3.71)	0.001	0.001
Creatinine	(mg/dL)	0.8	(0.15)	0.9	(0.13)	0.7	(0.09)	<0.001	<0.001
SBP	(mmHg)	116.6	(13.88)	119.2	(13.45)	114.1	(13.84)	<0.001	<0.001
DBP	(mmHg)	72.7	(11.20)	76.0	(10.64)	69.6	(10.81)	<0.001	<0.001
Pulse rate	(bpm)	70.6	(10.29)	70.8	(10.68)	70.3	(9.93)	0.548	0.704
CD4 cells	(count)	16,609.5	(6208.88)	16,433.2	(6301.24)	16,771.8	(6127.70)	0.494	0.159
CD8 cells	(count)	6092.7	(3185.58)	6125.7	(3176.89)	6062.2	(3198.10)	0.802	0.334
CD4/CD8	(ratio)	3.2	(1.51)	3.2	(1.60)	3.2	(1.41)	0.767	0.154

BMI, body mass index; HbA1c, hemoglobin A1c; HDL, high-density lipoprotein cholesterol; LDL, low-density lipoprotein cholesterol; ALT, alanine transaminase; AST, aspartate transaminase; γ-GTP, γ-glutamyl transferase; SBP, systolic blood pressure; DBP, diastolic blood pressure; SD, standard deviation.

**Table 2:** Characteristics of each CD8+ T cell in lymphocytes (%).

	All	(N = 632)	Male	(N = 303)	Female	(N = 329)	*t*-Test	Wilcoxon
Variables	Mean	(SD)	Mean	(SD)	Mean	(SD)	*p*	*p*
Total CD8+	19.1	(6.59)	18.9	(6.58)	19.3	(6.60)	0.487	0.488
Naïve in all subsets	34.6	(17.50)	34.3	(17.77)	34.8	(17.28)	0.721	0.468
TCM in all subsets	21.5	(9.37)	21.5	(9.74)	21.5	(9.03)	0.925	0.689
TEM in all subsets	30.1	(12.52)	31.2	(12.88)	29.1	(12.11)	0.036	0.031
TEMRA in all subsets	13.9	(11.69)	13.1	(10.40)	14.6	(12.74)	0.102	0.178
SA-βGal^high^ in total CD8+	65.5	(20.07)	65.7	(20.91)	65.2	(19.30)	0.737	0.456
SA-βGal^high^ in naïve	10.1	(9.27)	10.1	(9.55)	10.1	(9.02)	0.994	0.443
SA-βGal^high^ in TCM	53.1	(22.62)	53.0	(22.93)	53.2	(22.36)	0.908	0.882
SA-βGal^high^ in TEM	93.2	(7.72)	93.0	(8.21)	93.5	(7.24)	0.384	0.679
SA-βGal^high^ in TEMRA	96.3	(5.33)	96.3	(5.47)	96.3	(5.21)	0.918	0.617
PD-1+	9.8	(5.83)	10.5	(6.53)	9.2	(5.05)	0.007	0.031

SA-βGal, senescence-associated β-galactosidase; SD, standard deviation; TCM, central memory T cell; TEM, effector memory T cell; TEMRA, terminally differentiated effector memory T cells re-expressing CD45RA.

**Table 3:** Analysis of the correlation coefficient between age and each CD8+ T cell in lymphocytes (%).

	All				Male				Female			
Characteristics	Pearson	*p*	Spearman	*p*	Pearson	*p*	Spearman	*p*	Pearson	*p*	Spearman	*p*
Total CD8+	−0.119	0.003	−0.117	0.003	−0.157	0.006	−0.148	0.010	−0.084	0.126	−0.084	0.127
Naïve in all subsets	−0.308	<0.001	−0.320	<0.001	−0.326	<0.001	−0.348	<0.001	−0.291	<0.001	−0.297	<0.001
TCM in all subsets	0.098	0.014	0.085	0.033	0.148	0.010	0.122	0.034	0.048	0.388	0.046	0.406
TEM in all subsets	0.263	<0.001	0.259	<0.001	0.296	<0.001	0.287	<0.001	0.233	<0.001	0.233	<0.001
TEMRA in all subsets	0.102	0.010	0.114	0.004	0.053	0.362	0.112	0.052	0.140	0.011	0.117	0.034
SA-βGal^high^ in total CD8+	0.300	<0.001	0.314	<0.001	0.317	<0.001	0.348	<0.001	0.284	<0.001	0.282	<0.001
SA-βGal^high^ in naïve	0.254	<0.001	0.275	<0.001	0.300	<0.001	0.328	<0.001	0.210	<0.001	0.222	<0.001
SA-βGal^high^ in TCM	0.269	<0.001	0.272	<0.001	0.346	<0.001	0.350	<0.001	0.197	<0.001	0.196	<0.001
SA-βGal^high^ in TEM	0.169	<0.001	0.202	<0.001	0.175	0.002	0.228	<0.001	0.162	0.003	0.170	0.002
SA-βGal^high^ in TEMRA	0.148	<0.001	0.199	<0.001	0.131	0.023	0.184	0.001	0.164	0.003	0.211	<0.001

SA-βGal, senescence-associated β-galactosidase; TCM, central memory T cell; TEM, effector memory T cell; TEMRA, terminally differentiated effector memory T cells re-expressing CD45RA.

**Table 4:** Linear regression analysis of correlation between age and each CD8+ T cell in lymphocytes (%).

	Univairate					Multivariate				
Characteristics	β	95% CI			*p*	β	95% CI			*p*
Total CD8+	−0.151	−0.249	to	−0.053	0.003	−0.150	−0.248	to	−0.051	0.003
Naïve in all subsets	−1.034	−1.284	to	−0.784	<0.001	−1.038	−1.288	to	−0.788	<0.001
TCM in all subsets	0.175	0.036	to	0.315	0.014	0.176	0.036	to	0.316	0.014
TEM in all subsets	0.630	0.449	to	0.811	<0.001	0.633	0.453	to	0.814	<0.001
TEMRA in all subsets	0.228	0.054	to	0.403	0.010	0.229	0.054	to	0.403	0.010
SA-βGal^high^ in total CD8+	1.154	0.867	to	1.441	<0.001	1.156	0.869	to	1.444	<0.001
SA-βGal^high^ in naïve	0.452	0.317	to	0.586	<0.001	0.450	0.315	to	0.584	<0.001
SA-βGal^high^ in TCM	1.166	0.840	to	1.493	<0.001	1.163	0.836	to	1.490	<0.001
SA-βGal^high^ in TEM	0.249	0.135	to	0.363	<0.001	0.249	0.135	to	0.364	<0.001
SA-βGal^high^ in TEMRA	0.151	0.072	to	0.230	<0.001	0.151	0.072	to	0.230	<0.001

Multivariate: adjusted for sex and body mass index. CI, confidence interval; SA-βGal, senescence-associated β-galactosidase; TCM, central memory T cell; TEM, effector memory T cell; TEMRA, terminally differentiated effector memory T cells re-expressing CD45RA.

**Table 5:** Analysis of the correlation coefficient between the proportion of SA-βGal^high^ in total CD8+ T cells and each CD8+ T cell subset (%).

	All				Male				Female			
Characteristics	Pearson	*p*	Spearman	*p*	Pearson	*p*	Spearman	*p*	Pearson	*p*	Spearman	*p*
Naïve in all subsets	−0.915	<0.001	−0.928	<0.001	−0.925	<0.001	−0.937	<0.001	−0.906	<0.001	−0.919	<0.001
TCM in all subsets	−0.049	0.218	−0.092	0.021	−0.020	0.725	−0.062	0.281	−0.080	0.148	−0.113	0.041
TEM in all subsets	0.789	<0.001	0.797	<0.001	0.822	<0.001	0.828	<0.001	0.758	<0.001	0.773	<0.001
TEMRA in all subsets	0.565	<0.001	0.618	<0.001	0.582	<0.001	0.624	<0.001	0.565	<0.001	0.608	<0.001

SA-βGal, senescence-associated β-galactosidase; TCM, central memory T cell; TEM, effector memory T cell; TEMRA, terminally differentiated effector memory T cells re-expressing CD45RA.

**Table 6:** Linear regression analysis of correlation between the proportion of SA-βGal^high^ in total CD8+ T cells and each CD8+ T cell subset (%).

	Univairate					Multivariate				
Characteristics	β	95% CI			*p*	β	95% CI			*p*
Naïve in all subsets	−1.050	−1.081	to	−1.005	<0.001	−1.044	−1.082	to	−1.006	<0.001
TCM in all subsets	−0.105	−0.273	to	−0.062	0.218	−0.171	−0.331	to	−0.010	0.037
TEM in all subsets	1.265	1.188	to	1.342	<0.001	1.231	1.152	to	1.310	<0.001
TEMRA in all subsets	0.970	0.859	to	1.081	<0.001	0.932	0.826	to	1.039	<0.001

Multivariate: adjusted for age, sex, and body mass index. CI, confidence interval; SA-βGal, senescence-associated β-galactosidase; TCM, central memory T cell; TEM, effector memory T cell; TEMRA, terminally differentiated effector memory T cells re-expressing CD45RA.

## Data Availability

The data cannot be shared publicly because of ethical concerns.

## Supplementary Materials

The following supporting information can be downloaded at: https://www.mdpi.com/article/10.3390/ijms26188799/s1.
