# Supplementary material for: Correlation Between the Proportion of Senescence-Associated β-Galactosidase-Stained CD8+ T Cells and Age: A Cross-Sectional Study in Japan
Source: Int J Mol Sci. 2025 Sep 10;26(18):8799. doi: 10.3390/ijms26188799 (PMC12469844; doi:10.3390/ijms26188799)

Figure S1-1. Boxplots for the proportion of each CD8+ subset in all CD8+ T cell subsets.

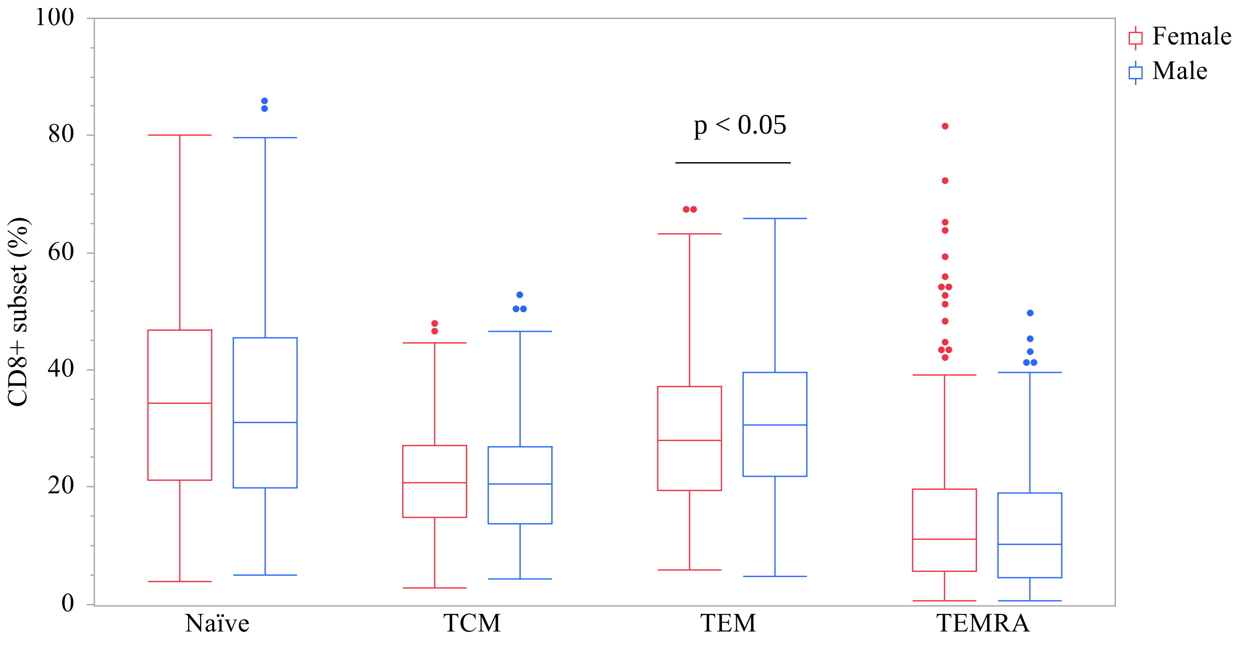

Figure S1-2. Boxplots for the proportion of SA-βGal^{high} in CD8+ T cells.

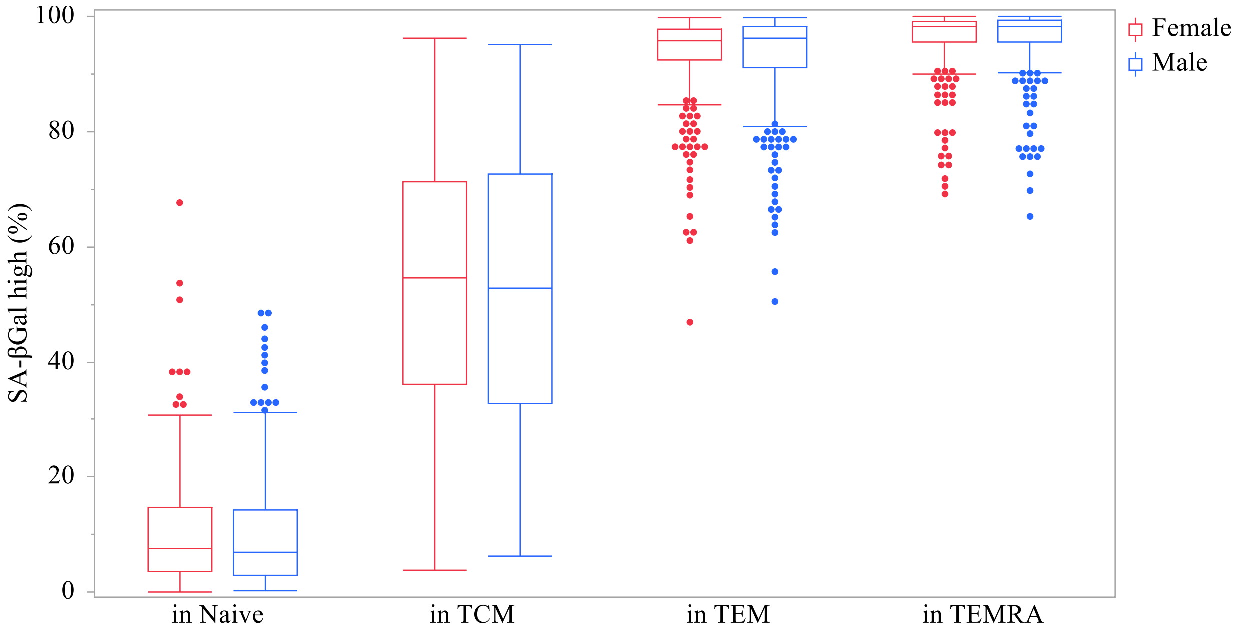

Figure S2-1. Correlation between the proportion of SA-βGal^{high} in naïve CD8+ T cells and age.

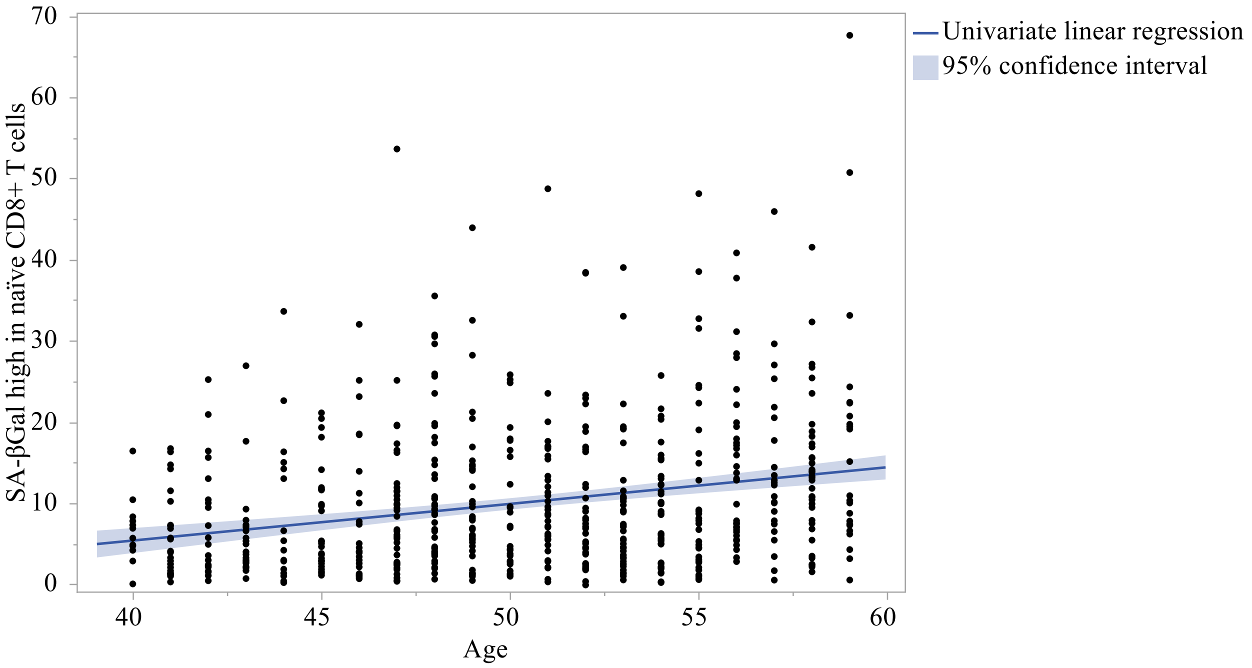

Figure S2-2. Correlation between the proportion of SA-βGal^{high} in TCM CD8+ T cells and age.

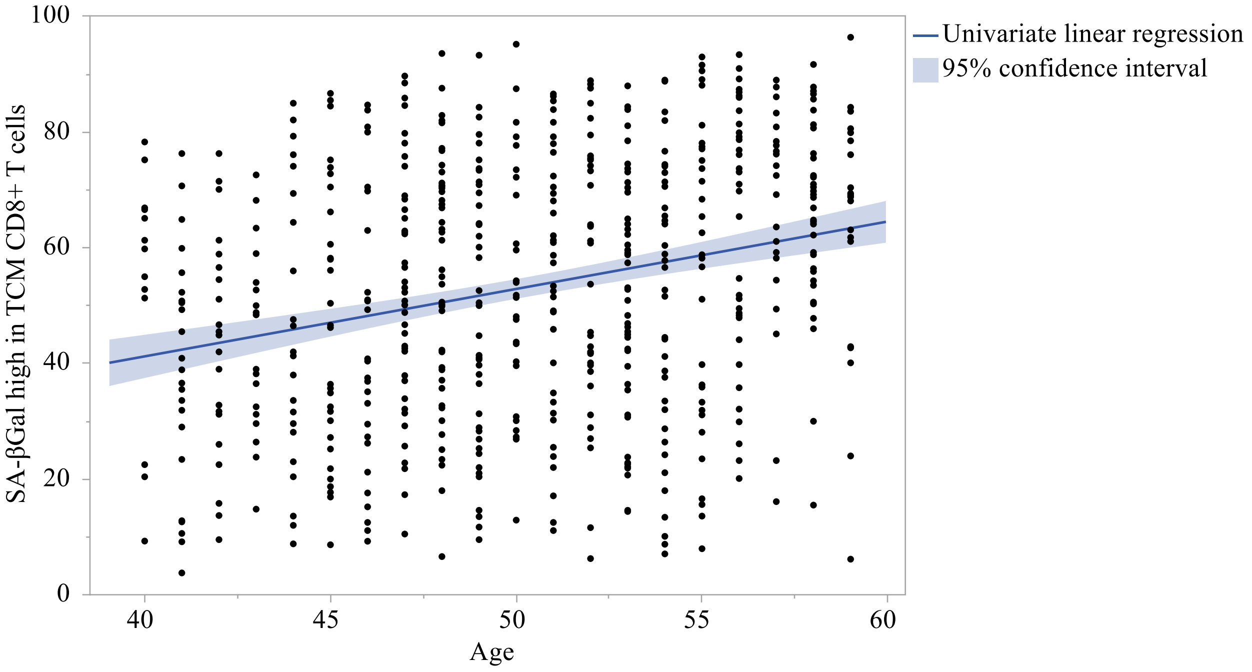

Figure S2-3. Correlation between the proportion of SA-βGal^{high} in TEM CD8+ T cells and age.

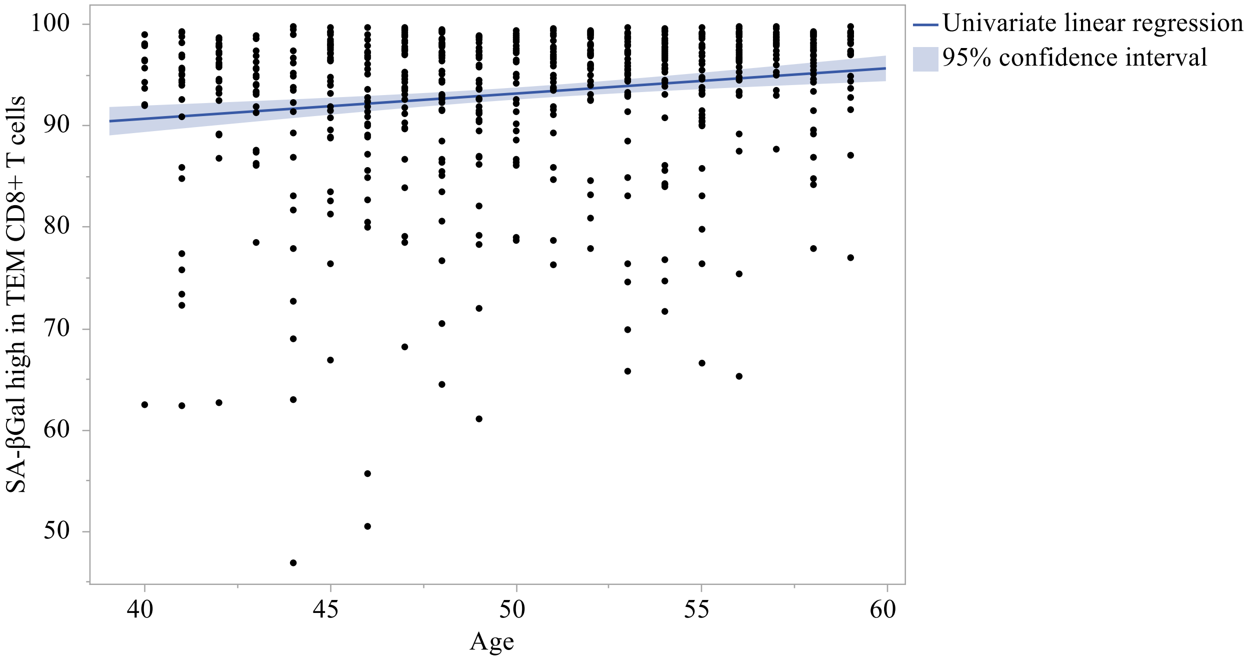

Figure S2-4. Correlation between the proportion of SA-βGal^{high} in TEMRA CD8+ T cells and age.

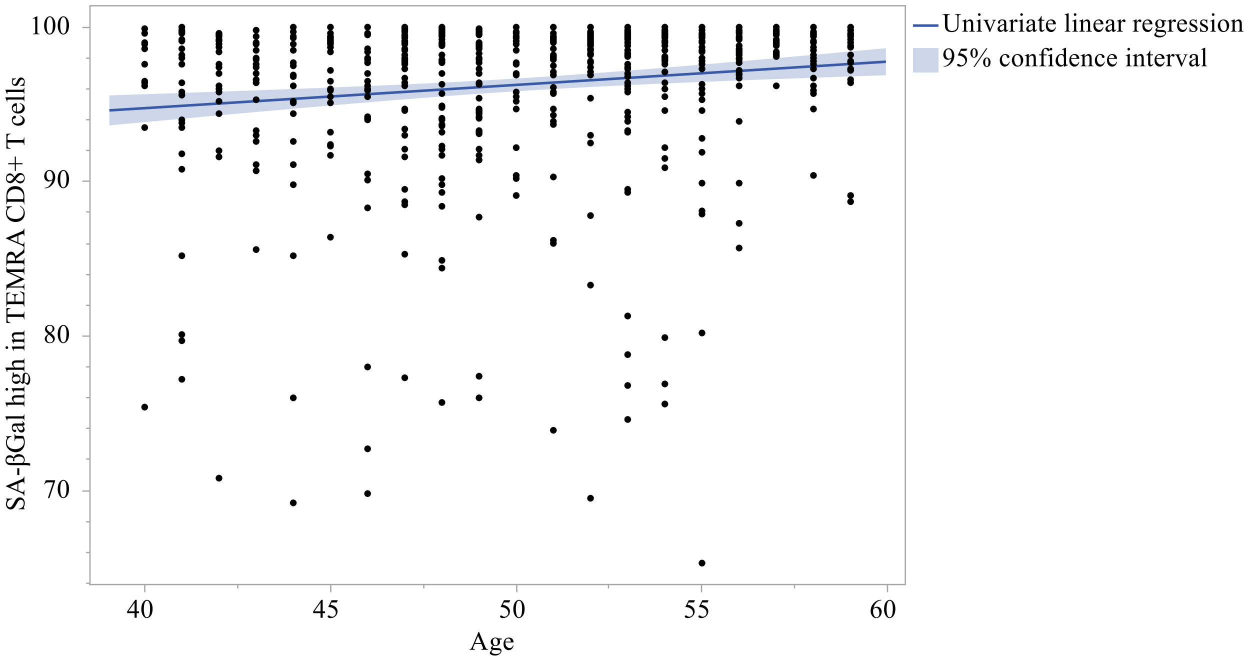

Figure S3-1. Correlation between the proportion of SA-βGal^{high} in total CD8+ T cells and naïve CD8+ T cells.

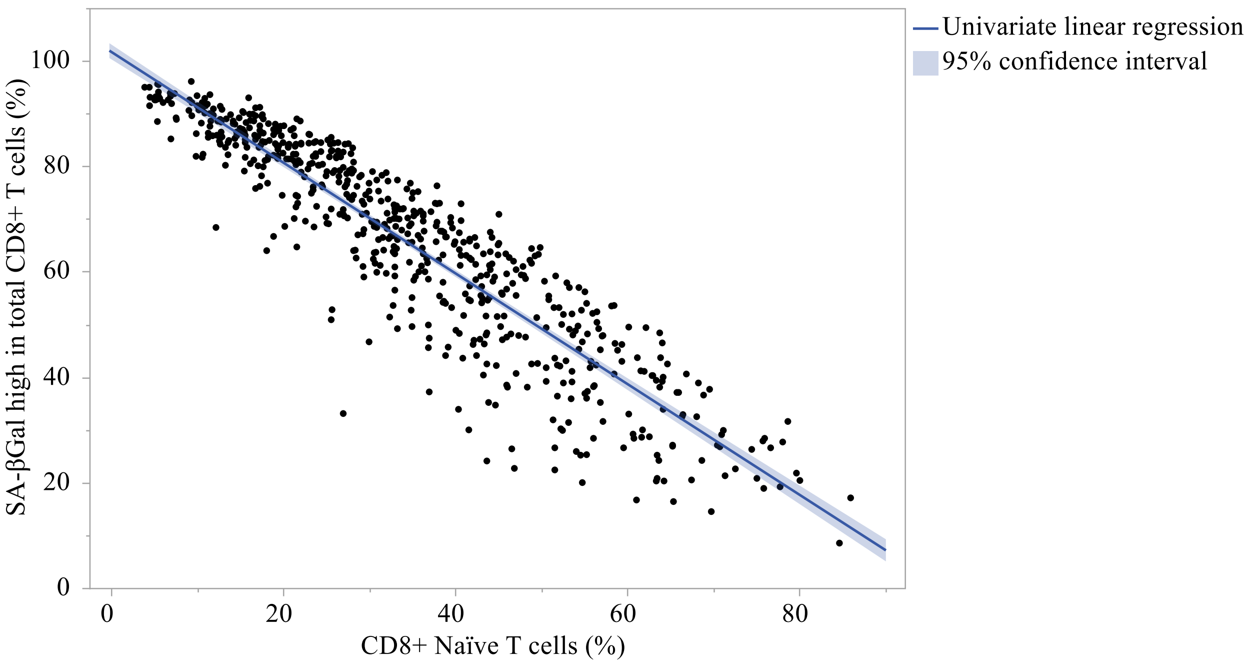

Figure S3-2. Correlation between the proportion of SA-βGal^{high} in total CD8+ T cells and TCM CD8+ T cells.

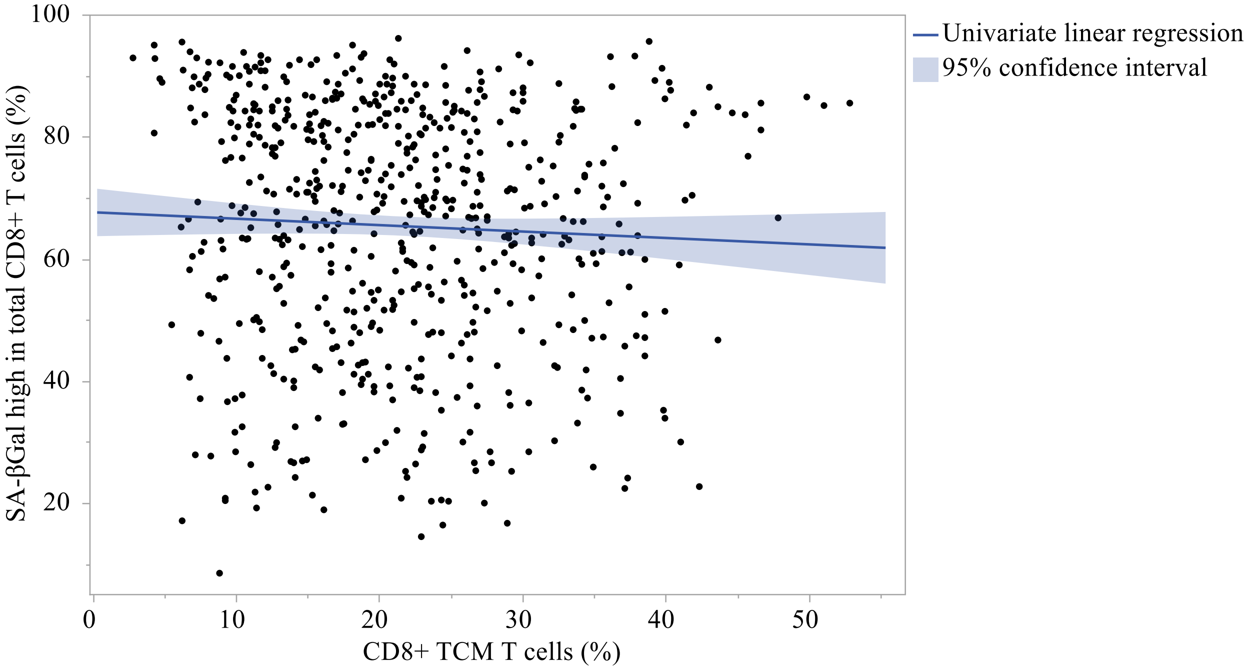

Figure S3-3. Correlation between the proportion of SA-βGal^{high} in total CD8+ T cells and CD8+ TEM T cells.

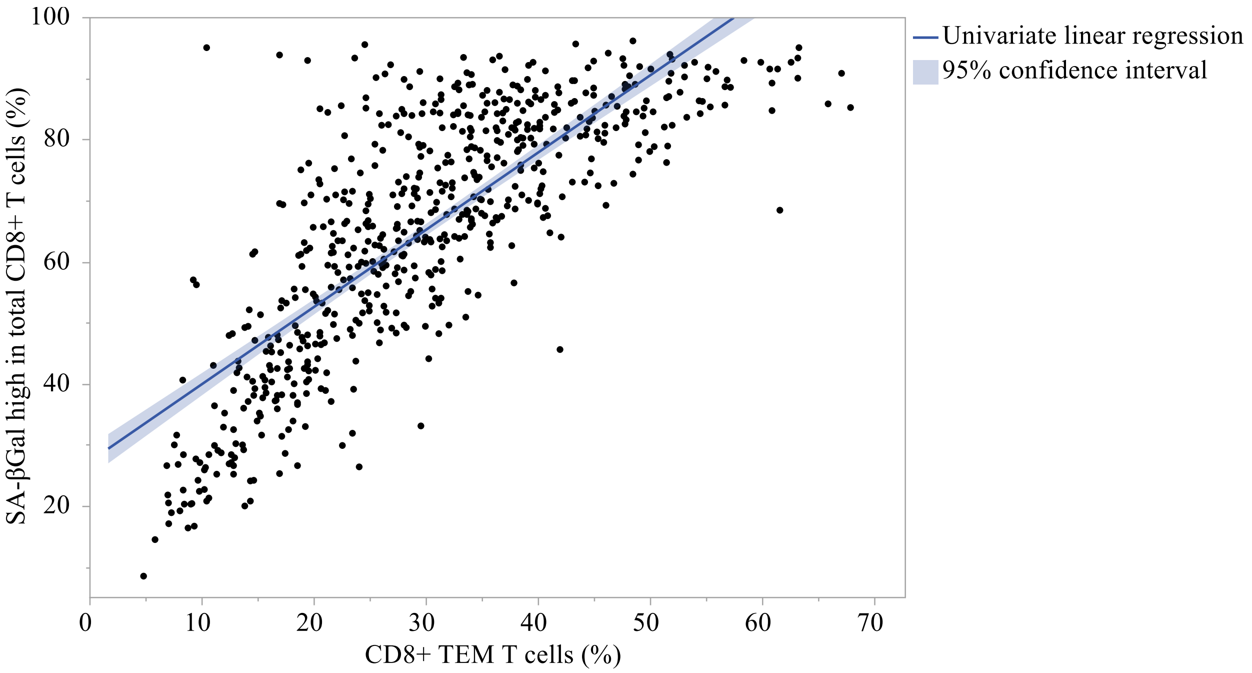

Figure S3-4. Correlation between the proportion of SA-βGal^{high} in total CD8+ T cells and CD8+ TEMRA T cells.

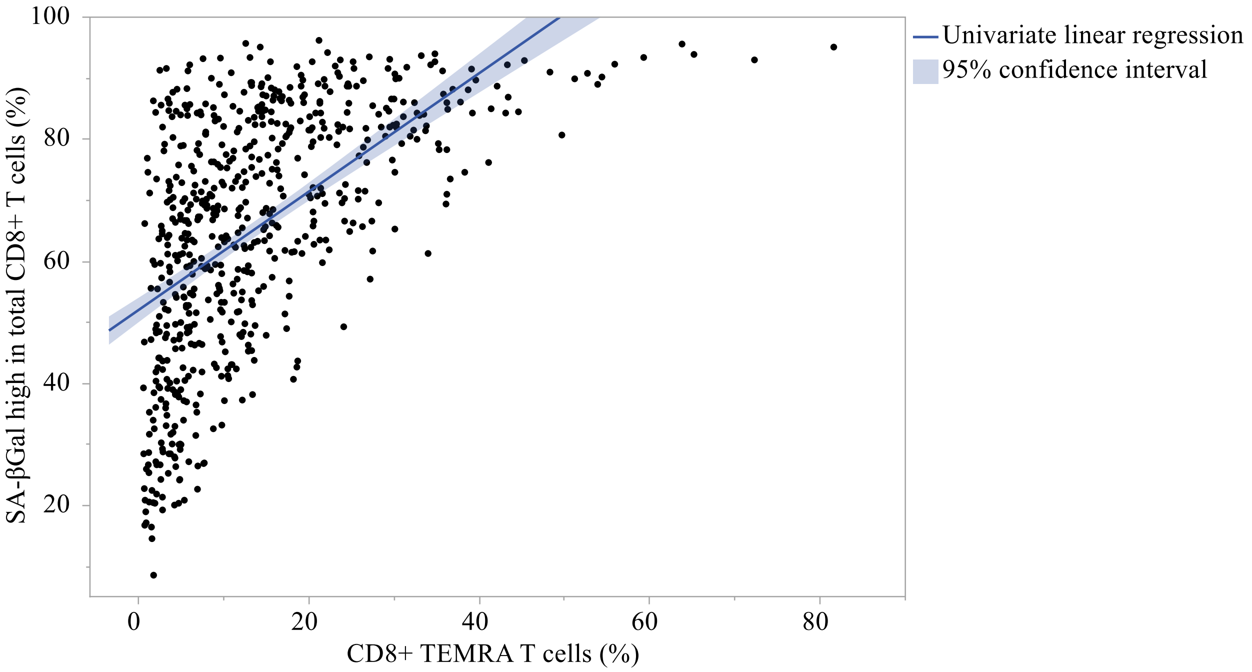

Supplement: Supplementary file 1 [file ijms-26-08799-s001.zip › ijms-3803006_Supplementary file1.pdf]
