# Supplementary material for: Correlation Between the Proportion of Senescence-Associated β-Galactosidase-Stained CD8+ T Cells and Age: A Cross-Sectional Study in Japan
Source: Int J Mol Sci. 2025 Sep 10;26(18):8799. doi: 10.3390/ijms26188799 (PMC12469844; doi:10.3390/ijms26188799)
Supplement: Supplementary file 1 [file ijms-26-08799-s001.zip › ijms-3803006_Supplementary file2.pdf]

Flow cytometric gating strategy.

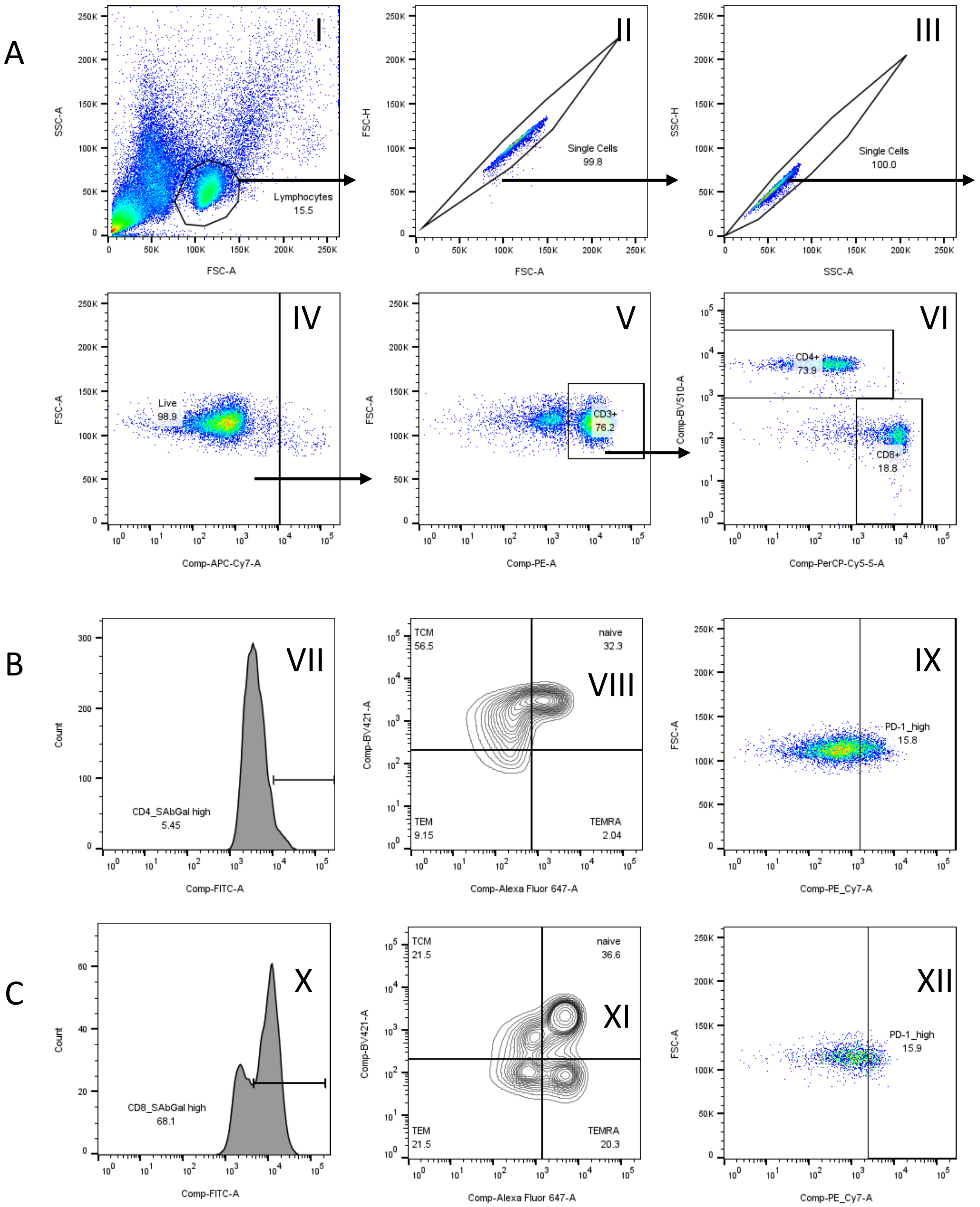

First, the gating strategy for selecting CD4-positive and CD8-positive cells from PBMCs is shown (A). The lymphocyte fraction of PBMCs is separated by SSC-A and FSC-A (I). Next, single cells are identified by plotting FSC-A vs. FSC-H (II) and then SSC-A vs. SSC-H (III). Dead cells are excluded from the single cells by gating on APC-Cy7-negative cells (IV). PE-positive cells are then identified as CD3-positive cells (V). Among the CD3-positive cells, BV510-positive cells are identified as CD4-positive cells, and PerCP-Cy5.5-positive cells are identified as CD8-positive cells (VI). Next, further gating strategies are shown for CD4-positive cells (B) and CD8-positive cells (C). SA-βGal^{high} expressing senescent cells are identified by gating on cells with high FITC fluorescence intensity (VII, X). CD8+ cells have a distinctive double-peaked pattern, and senescent cells are identified based on the valley between the two peaks. T cell subsets are classified as follows: Alexa Fluor 647-positive and BV421-positive cells are naïve cells, Alexa Fluor 647-negative and BV421-positive cells are TCM cells, Alexa Fluor 647-negative and BV421-negative cells are TEM cells, and Alexa Fluor 647-positive and BV421-negative cells are TEMRA cells (VIII, XI). Finally, PD-1-high expressing cells are identified by gating on cells with strong PE-Cy7 fluorescence intensity (IX, XII).
